# Enhanced Water Absorbency and Water Retention Rate for Superabsorbent Polymer via Porous Calcium Carbonate Crosslinking

**DOI:** 10.3390/nano13182575

**Published:** 2023-09-17

**Authors:** Yixin Jiao, Tongming Su, Yongmei Chen, Minggui Long, Xuan Luo, Xinling Xie, Zuzeng Qin

**Affiliations:** 1 School of Chemistry and Chemical Engineering, Guangxi University, Nanning 530004, China; jiao961216jiao@163.com (Y.J.); sutm@gxu.edu.cn (T.S.); luoxuan@gxu.edu.cn (X.L.); xinlingxie@gxu.edu.cn (X.X.); 2 Guilin Zhuorui Food Ingredients Co., Ltd., Guilin 541001, China; lilychym-123@163.com (Y.C.); longmg@126.com (M.L.)

**Keywords:** porous calcium carbonate, crosslinking, chelation, composite superabsorbent polymer

## Abstract

To improve the water absorbency and water-retention rate of superabsorbent materials, a porous calcium carbonate composite superabsorbent polymer (PCC/PAA) was prepared by copolymerization of acrylic acid and porous calcium carbonate prepared from ground calcium carbonate. The results showed that the binding energies of C–O and C=O in the O 1*s* profile of PCC/PAA had 0.2 eV and 0.1–0.7 eV redshifts, respectively, and the bonding of –COO^−^ groups on the surface of the porous calcium carbonate led to an increase in the binding energy of O 1*s*. Furthermore, the porous calcium carbonate chelates with the –COO^−^ group in acrylic acid through the surface Ca^2+^ site to form multidirectional crosslinking points, which would increase the flexibility of the crosslinking network and promote the formation of pores inside the PCC/PAA to improve the water storage space. The water absorbency of PCC/PAA with 2 wt% porous calcium carbonate in deionized water and 0.9 wt% NaCl water solution increased from 540 g/g and 60 g/g to 935 g/g and 80 g/g, respectively. In addition, since the chemical crosslinker *N*,*N*′-methylene bisacrylamide is used in the polymerization process of PCC/PAA, *N*,*N*′-methylene bisacrylamide and porous calcium carbonate enhance the stability of the PCC/PAA crosslinking network by double-crosslinking with a polyacrylic acid chain, resulting in the crosslinking network of PCC/PAA not being destroyed after water absorption saturation. Therefore, PCC/PAA with 2 wt% porous calcium carbonate improved the water-retention rate by 244% after 5 h at 60 °C, and the compressive strength was approximately five-times that of the superabsorbent without porous calcium carbonate.

## 1. Introduction

Superabsorbent polymers are functional polymeric materials with physically or chemically crosslinked 3D hydrophilic networks. These materials can absorb water hundreds, even thousands of times their weight and retain large amounts of water within their network structures [1,2,3] and with mechanical strength after absorbing water [4]. Superabsorbent polymers with excellent water absorbency and water-retention rates are widely used in the fields of hygienic products [5], soil-water-retention agents [6], wastewater treatment agents [7], and building materials [8]. However, the complex environment affects the actual water-absorption process, such as the temperature, pressure, pH, and ionic strength of the solution [9,10]. Superabsorbent polymers still have the problems of high water absorbency and water retention and low salt tolerance, which limits their application in the field of hygienic products.

Crosslinked network structures are critical to the superabsorbent polymer’s water absorbency [11], and a flexible network structure can provide a large amount of free space for the superabsorbent polymer to hold large amounts of water. Currently, the network structure of the superabsorbent polymer is modified by constructing a double-network [12,13], an inorganic/organic composite network [14,15,16], and an interpenetrating polymer network [17] to improve the water absorbency, salt tolerance, water-retention rate, and reusability of the superabsorbent polymer. Among the different superabsorbent-polymer-enhancement methods, the incorporation of inorganic filler could not only reduce the production cost, but also improve the superabsorbent polymer crosslinking network structure while increasing the water absorbency and water-retention rate [18,19,20,21,22].

Calcium carbonate is widely used in polymer fillers because of its simple production procedure and low-cost characteristics [23] and can be used to improve the superabsorbent polymer crosslinking network structure. In a previous report, our group prepared a bagasse cellulose composite superabsorbent polymer using modified nanocalcium carbonate and *N*,*N*′-methylene bisacrylamide (MBA) as a double-crosslinker, demonstrating that the addition of modified nanocalcium carbonate facilitates the formation of a stable network and enhances the water absorbency of the polymer in deionized water, as well as in 0.9 wt% NaCl water solution [24]. In addition, compared with other inorganic fillers, calcium carbonate is a good filler in superabsorbent polymers because the distribution of Ca^2+^ ions on the surface of calcium carbonate crystals [25] allows calcium carbonate to crosslink polymeric chains through chelation interactions with the –COOH groups [26]. This chelation can be used in superabsorbent polymer network construction to obtain more-flexible crosslinking networks [27]. However, although common calcium carbonate, such as ground calcium carbonate, has cost advantages, the small specific surface area makes it difficult to provide enough sites for crosslinking polymer chains. Nanocalcium carbonate is small in size and large in surface area. However, limited by the size effect of nanoparticles, the particles can easily combine [28] and cause uneven dispersion in the polymer network, destroying the network structure of the superabsorbent polymer. Finding an inorganic filler that can significantly improve the water absorbency and water-retention rate of a superabsorbent polymer is still challenging.

Porous calcium carbonate possesses a rich pore structure and large specific surface area [29,30,31,32], which enhance the interaction with the superabsorbent polymer network and improve the crosslinking network structure of the superabsorbent polymer as an inorganic crosslinking component [33]. The microparticles have better dispersion than nanocalcium carbonate. In this work, ground calcium carbonate obtained from the grinding of natural minerals was used as the raw material, and the precipitated calcium carbonate was coated on the surface of the ground calcium carbonate to obtain porous calcium carbonate. Porous calcium carbonate and MBA were used as bifunctional crosslinkers to obtain a composite superabsorbent polymer (PCC/PAA). The influence of porous calcium carbonate crosslinking on the structure, water absorbency, water-retention rate, compressive strength, and reswelling water absorbency of PCC/PAA was studied.

## 2. Materials and Methods

### 2.1. Preparation of Porous Calcium Carbonate and PCC/PAA

Preparation of porous calcium carbonate: The ground calcium carbonate (3000-mesh, specific surface area of 1.6 m^2^/g, Guilin Zhuorui Food Ingredients Co., Ltd., Guilin, China) was modified using polyacrylic acid (Mv approximately 250,000, Guangdong Wengjiang Reagent Co., Ltd., Shaoguan, China) to coat the synthetic calcium carbonate on the surface of the ground calcium carbonate, and further porous calcium carbonate was obtained. Ground calcium carbonate was modified according to the literature method to obtain modified ground calcium carbonate [34], in which the dosage of polyacrylic acid was 10% of the mass of ground calcium carbonate. The brief procedure of the ground calcium carbonate modified with polyacrylic acid was as follows: The 20 wt% ground calcium carbonate slurry with polyacrylic acid was mixed and reacted under mechanical stirring at 400 r/min and 90 °C for 3 h, followed by solid–liquid separation, dried at 60 °C for 24 h, pulverized, and passed through a 200-mesh sieve. Then, 0.500 g of modified ground calcium carbonate was mixed with 40 mL of 0.05 mol/L CaCl_2_ (AR, Chengdu Kelong Chemical Co., Ltd., Chengdu, China) solution and stirred for 30 min to obtain the mixed slurry. Sodium dodecyl sulfate (0.576 g, AR, Chengdu Jinshan Chemical Reagent Co., Ltd., Chengdu, China) was added to 40 mL of 0.05 mol/L Na_2_CO_3_ (AR, Chengdu Kelong Chemical Co., Ltd.) solution, and then, the Na_2_CO_3_ solution was added to the mixed slurry containing modified ground calcium carbonate at 4 mL/min. The reaction was stirred at 25 °C and 200 r/min for 10 min, washed with ethanol (Sichuan Xilong Science Co., Ltd., Chengdu, China) and deionized water, dried at 60 °C for 12 h, and passed through a 200-mesh sieve to obtain porous calcium carbonate (the content of polyacrylic acid was 6 wt%).

Preparation of PCC/PAA: The acrylic acid monomer (5.000 g) (AR, Shanghai Macklin Biochemical Co., Ltd., Shanghai, China) was neutralized with a 21 mL of 2 mol/L NaOH (AR, Sichuan Xilong Science Co., Ltd.) solution in an ice water bath and then added to 40 mL of deionized water containing porous calcium carbonate (relative to 2–8 wt% of the total mass of the monomer) with 0.025 g of MBA (AR, Shanghai Macklin Biochemical Co., Ltd.). After increasing the temperature to 60 °C, 0.120 g of potassium persulfate (KPS) (AR, Shanghai Macklin Biochemical Co., Ltd.) was added to initiate polymerization, and the reaction was allowed to proceed for 3 h at a constant temperature. After the reaction, the product was soaked in absolute ethanol for 24 h, cleaned, and dried to a constant weight at 60 °C. PCC (2 wt%)/PAA-, PCC (4 wt%)/PAA-, PCC (6 wt%)/PAA-, and PCC (8 wt%)/PAA-based superabsorbents with 2 wt%, 4 wt%, 6 wt%, and 8 wt% porous calcium carbonate, respectively, were obtained. For comparison, a PAA-based superabsorbent without porous calcium carbonate was prepared by free radical polymerization of acrylic acid monomers. PCC/PAA particles in the 40–60-mesh range were selected for water absorbency detection.

### 2.2. Characterization Method

The X-ray diffraction (XRD) patterns were recorded on a Rigaku D/MAX 2500 V X-ray diffractometer (Rigaku, Tokyo, Japan) with Cu Kα radiation used for phase detection. The microstructure of the samples was obtained by a Hitachi SU8220 scanning electron microscope (Hitachi Limited, Tokyo, Japan). Fourier transform infrared (FT-IR) spectra were obtained by a Tensor II FT-IR spectrometer (Bruker, Mannheim, Germany) using KBr pellets in the range of 4000–400 cm^−1^ to investigate the chemical nature of the particles. The pore volume, BET-specific surface area, and BJH pore size of the porous calcium carbonate were determined by N_2_ adsorption–desorption measurements using an ASAP 2020 (Micrometrics, Norcross, GA, USA). The X-ray photoelectron spectroscopy (XPS) profiles were recorded on a Thermo Scientific K-Alpha (Thermo Fisher Scientific, Waltham, MA, USA). Thermogravimetric (TG-DTG) analysis was performed on a Thermo Plus EVO2 thermogravimetric analyzer (Rigaku, Japan) under a N_2_ atmosphere at 10 °C/min from 30 °C to 800 °C. The PCC/PAA sample saturated with water absorption was cut into cuboid shapes (the length, width, and height were 3 cm, 3 cm, and 2 cm, respectively, and the weight was approximately 18 g), and the compressive test of samples was performed using a universal electronic material testing machine (Instron, Norwood, MA, USA) at a compression rate of 5 mm/min.

### 2.3. Measurement of Water Absorbency

To measure the water absorbency of the superabsorbent sample over time in distilled water and the 0.9 wt% NaCl water solution, 0.01 g of dry sample was immersed in 500 mL of deionized water or 0.9 wt% NaCl water solution for different durations (20 s, 50 s, 100 s, 200 s, 300 s, 600 s, 900 s, 1200 s, 1500 s, 1800 s, 2100 s, and 2400 s). After the specified period, the water-absorbed sample was filtered with a 100-mesh stainless-steel sieve and drained for 10 min to remove unabsorbed water. Then, the weight of the water-absorbed sample was measured, and its water absorbency at a particular time interval was calculated by Equation (1).

To measure the salt tolerance and pH sensitivity of the superabsorbent sample, the water absorbency of the as-obtained superabsorbent samples in different concentrations of NaCl water solutions, CaCl_2_ solutions, and different pH solutions were tested by the gravimetric method. Typically, 0.01 g of the dry sample was immersed into different concentrations of the 500 mL NaCl water solutions, CaCl_2_ solutions, and various pH solutions (0.1 mol/L HCl and 0.1 mol/L NaOH were diluted with deionized water to obtain solutions of different pH values) until no more change in the weight of the superabsorbent sample was observed. It was eventually filtered and drained for 10 min to remove unabsorbed water from the water-absorbed sample by using a 100-mesh stainless-steel sieve and then weighed accurately. The water absorbency can be calculated by Equation (1).

(1) $$\mathrm{Water}\;\mathrm{absorbency}\left(g/g\right)=\frac{W_{t}-W_{d}}{W_{d}}$$

where *W*_t_ and *W*_d_ represent the weight of the water-absorbed superabsorbent sample at time t and the initial weight of the dry sample, respectively, g.

To determine the water retention capability, the as-obtained superabsorbent samples, after reaching their equilibrium swelling at 25 °C, were collected from the deionized water and used in the following water-retention experiments. In a water-retention experiment, the water-absorbed superabsorbent samples were placed in an oven at 20 °C, 40 °C, and 60 °C for 5 h, respectively. Then, the samples were removed, and the weight was measured every hour for 5 h of continuous measurement. The oven’s upper set of exhaust valves was used to control the humidity of the oven to maintain the same range when the water-retention rate was measured to ensure that all the water retention tests were performed under the same conditions. The water retention ratio was calculated by Equation (2).

(2) $$\mathrm{Water}\;\mathrm{retention}\;\mathrm{ratio}\left(\%\right)=\frac{W_{t}-W_{d}}{W_{s}-W_{d}}\times 100$$

where *W*_d_, *W*_t_, and *W*_s_ are the weights of the dry sample, the water-absorbed sample at time t, and the water-absorbed sample at the starting time, respectively, g.

## 3. Results and Discussion

### 3.1. Characterization of PCC/PAA

**Figure 1:**
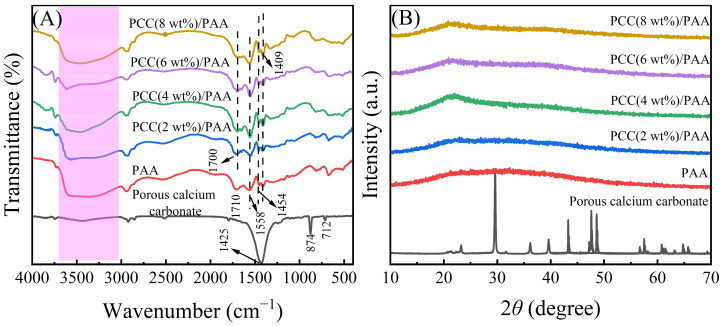
FT-IR spectra (**A**) and XRD patterns (**B**) of porous calcium carbonate, PAA, and PCC/PAA with 2–8 wt% porous calcium carbonate.

Porous calcium carbonate crosslinked the PAA copolymer to obtain a novel composite superabsorbent polymer PCC/PAA. The composite superabsorbent material was characterized to understand the crosslinking mechanism between porous calcium carbonate and PCC/PAA. Figure 1A shows the FT-IR spectra of porous calcium carbonate, PAA, and PCC/PAA with 0–8 wt% porous calcium carbonate. For porous calcium carbonate, the band at 1425 cm^−1^ corresponds to the C–O antisymmetric stretching vibration, the band at 874 cm^−1^ corresponds to the CO_3_^2−^ out-plane bending vibrations of CO_3_^2−^, and a relatively weak absorption band at 712 cm^−1^ corresponding to the CO_3_^2−^ in-plane bending vibrations was attributed to calcite [35]. In the FT-IR spectrum of PAA, the broadband at 3500 cm^−1^ (the pink highlight in the Figure 1A) was attributed to –OH in the polymer. The band at 1708 cm^−1^ corresponding to the C=O stretching vibration implies the presence of the –COOH group [36]. The N–H bending vibration was observed at 1558 cm^−1^, indicating that the crosslinker MBA was incorporated into the polymer backbone and crosslinked the polymer chains [37]. These characteristic bands indicated that the acrylic acid monomers were successfully polymerized into the PAA copolymer. The band at 1454 cm^−1^ was attributed to the –COO^−^ symmetrical stretching vibration. The O–H bending vibration at 1409 cm^−1^ was attributed to the –COOH group.

Additionally, the band of the C=O bond at 1710 cm^−1^ of PAA was shifted to 1700 cm^−1^ of PCC/PAA with 2–8 wt% porous calcium carbonate with an offset of 10 cm^−1^. The reason for the characteristic band shift of C=O was attributed to the combination of the polyacrylic acid chain and porous calcium carbonate, which balances the electron cloud of chemical bonds, increases the bond length of C=O, and decreases the vibration frequency, leading to a redshift of the C=O vibration band [38]. It is clear that, during the polymerization of PCC/PAA, the porous calcium carbonate was involved in the construction of the crosslinking network as an inorganic crosslinking component through the crosslinking of the polyacrylic chain [39] and formed a double-crosslinking network with the chemical crosslinker *N*,*N*′-methylene diacrylamide.

Figure 1B shows the XRD patterns of porous calcium carbonate, PAA, and PCC/PAA with 2–8 wt% porous calcium carbonate. For porous calcium carbonate, the characteristic peaks of calcite were detected at 23.0°, 29.5°, 31.5°, 36.0°, 39.5°, 43.3°, 47.5°, 48.6°, and 57.5°, corresponding to the (012), (104), (006), (110), (113), (202), (018), (116), and (211) crystal planes of calcite, respectively [40]. After copolymerization of the porous calcium carbonate with acrylic acid, the characteristic peak of the porous calcium carbonate in PCC/PAA disappeared as the porous calcium carbonate was encapsulated by the polyacrylic acid chains, indicating that the porous calcium carbonate was involved in the construction of the polymer crosslinking network [41]. In addition, PAA and PCC/PAA showed a broad peak at 22.0°, which was attributed to the crystal structure formed by the crosslinking of the polymer chains [42]. As the content of porous calcium carbonate increased to 4 wt%, the increased intensity of the peak at 22.0° implied that the porous calcium carbonate enhanced the crosslinking of the polyacrylic chains to ensure that the crosslinked network structure of PCC/PAA was not disrupted when fully extended [43]. However, as the porous calcium carbonate content increased from 4 wt% to 8 wt%, the peak intensity at 22.0° decreased, which was attributed to the increased crosslinking points, shortening the polyacrylic chain and inhibiting crosslinking.

**Figure 2:**
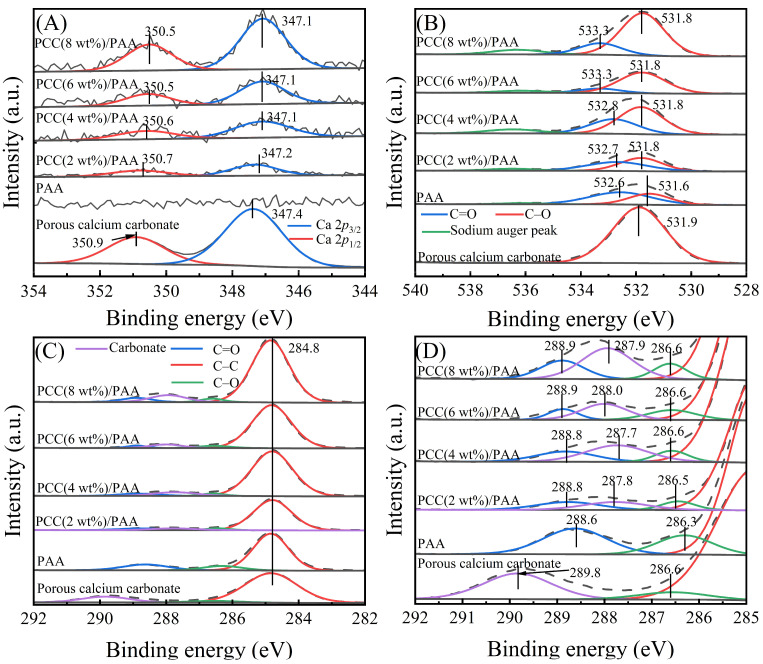
XPS profiles of Ca 2*p* (**A**), O 1*s* (**B**), and C 1*s* (**C**) and their magnified binding energies from 292–285 eV (**D**) for porous calcium carbonate, PAA, and PCC/PAA with 2–8 wt% porous calcium carbonate.

XPS measurements were carried out to investigate the change in the chemical bonds of the C 1*s*, O 1*s*, and Ca 2*p* elements before and after introducing porous calcium carbonate. Figure 2A shows the XPS profiles of Ca 2*p* for the porous calcium carbonate, PAA, and PCC/PAA with 2–8 wt% porous calcium carbonate. Compared to PAA, the new peak in PCC/PAA was attributed to the porous calcium carbonate in the crosslinked network [44]. Compared with the Ca 2*p* profile of pure porous calcium carbonate, the binding energies of Ca 2*p*_3/2_ and Ca 2*p*_1/2_ in PCC/PAA with 2–8 wt% porous calcium carbonate were shifted to lower energy levels by 0.2–0.3 eV and 0.2–0.4 eV, respectively. The results indicated that the –COO^−^ group chelates with Ca^2+^, and the electron cloud is transferred from the –COO^−^ group to the porous calcium carbonate surface, which increases the electron cloud density around Ca^2+^, and the electromagnetic shielding effect of the inner shell electron is enhanced, which leads to the binding energy shifting to a lower energy level [45].

Figure 2B shows the O 1*s* profiles of porous calcium carbonate, PAA, and PCC/PAA with 2–8 wt% porous calcium carbonate. For PAA, the two peaks at 531.6 eV and 532.6 eV correspond to C–O and C=O, respectively. However, in PCC (2 wt%)/PAA, the binding energies of C–O and C=O shifted to 531.8 eV and 532.7 eV, respectively, which moved toward higher energy levels by 0.2 eV and 0.1 eV, respectively, and continuously shifted to higher energy levels as the content of porous calcium carbonate increased. In addition, as shown in Figure 2C,D, the C 1*s* profiles of PAA consisted of three characteristic peaks, C–C, C–O, and C=O, located at binding energies of 284.8 eV, 286.3 eV, and 288.6 eV, respectively. However, in PCC/PAA with 2–8 wt% porous calcium carbonate, the binding energies of C–O and C=O shifted toward higher energy levels by 0.2–0.3 eV, respectively. The shift in the binding energy of C–O and C=O was attributed to the decrease in electron cloud density caused by the bonding of –COO^−^ on the porous calcium carbonate surface [46], confirming the chelation interaction between –COO^−^ and Ca^2+^ on the porous calcium carbonate surfaces [47]. In addition, the peak of carbonate belonging to the porous calcium carbonate was detected at 287.7–288.0 eV for PCC/PAA with different porous calcium carbonate contents, indicating that porous calcium carbonate exists in the crosslinked network of PCC/PAA in the form of inorganic crosslinking components.

**Figure 3:**
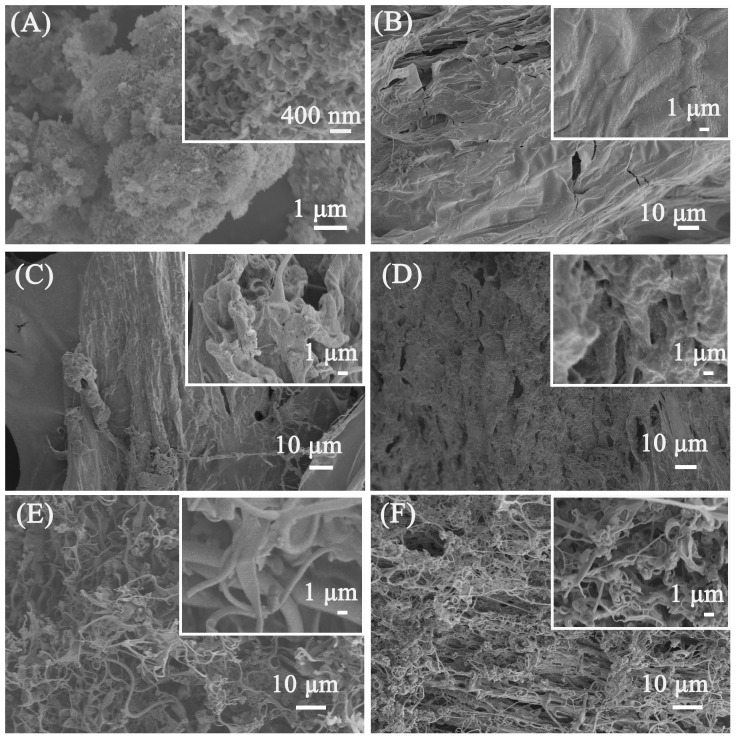
SEM images of porous calcium carbonate (**A**), PAA (**B**), PCC (2 wt%)/PAA (**C**), PCC (4 wt%)/PAA (**D**), PCC (6 wt%)/PAA (**E**), and PCC (8 wt%)/PAA (**F**).

SEM was used to study the morphology of the porous calcium carbonate for crosslinking polymers. The irregular morphology of the porous calcium carbonate can be observed in Figure 3A, and it can be observed that the surface of the porous calcium carbonate had pore channels formed by the aggregation of 200 nm flakes, which was attributed to the regulatory effect of sodium dodecyl sulfate on the morphology of the calcium carbonate. Through the N_2_ adsorption–desorption test, the BET specific surface area of the porous calcium carbonate can reach 48.1 m^2^/g, indicating that the surface of the porous calcium carbonate can provide Ca^2+^ sites to crosslink the polymer to form a firm 3D crosslink network. Figure 3B–F show the SEM images of PAA and PCC/PAA with 2–8 wt% porous calcium carbonate. As shown in Figure 3B, the PAA has a smooth surface without adding porous calcium carbonate. The dense surface of the PAA may lead to the blocking of water infiltration into the polymer network after polymer particles come into contact with water [48]. As shown in Figure 3C, when 2 wt% porous calcium carbonate was added, the PCC (2 wt%)/PAA morphology became rough. It showed approximately 0.5–1 μm pores, facilitating increased contact with water. The reason was that, in the process of PCC/PAA polymerization, when the total amount of monomer is constant, the increase in crosslink centers, which are generated by the rigid porous calcium carbonate particles between the polyacrylic acid chains, weakens the chain entanglement caused by hydrogen bond interactions between the polymer chains in the 3D network of PCC/PAA [49]. With the increase in porous calcium carbonate content to 4 wt%, the morphology of PCC (4 wt%)/PAA showed pores of approximately 1–5 μm, which act as channels for the diffusion of water into the polymer interior. However, when the porous calcium carbonate content reached 6–8 wt%, PCC (6 wt%)/PAA and PCC (8 wt%)/PAA consisted of an entangled filamentary network with gaps of approximately 1–10 μm. The reason is that, in the PCC/PAA polymerization process, porous calcium carbonate is used as an inorganic crosslinking component. The increase in its content increases the crosslinking points of the polyacrylic acid chain and shortens the molecular chain, making PCC/PAA show a loose structure, which is not conducive to water retention within the PCC/PAA crosslinking network.

**Figure 4:**
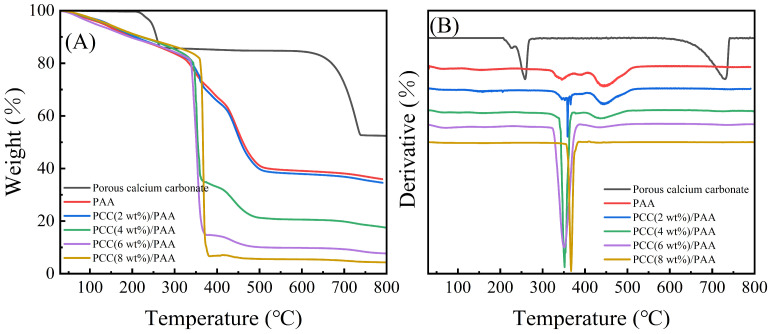
TG (**A**) and DTG (**B**) curves for porous calcium carbonate, PAA, and PCC/PAA with 2–8 wt% porous calcium carbonate.

The thermal stability of porous calcium carbonate, PAA, and PCC/PAA with 2–8 wt% porous calcium carbonate was evaluated, and the TG-DTG curves are shown in Figure 4. Figure 4A shows that the porous calcium carbonate had a weight loss of 47.48% from 30–800 °C, and the thermal decomposition starting at 205 °C was attributed to the removal of the –COOH groups in the polyacrylic acid modified on the surface of the ground calcium carbonate during the preparation [50]. The weight loss in the temperature range of 235–420 °C was attributed to the thermal decomposition of the polyacrylic acid backbone and the thermal decomposition of sodium dodecyl sulfate adsorbed on the surface of the calcium carbonate [51], and there was a total weight loss of approximately 14.94% at 205–420 °C. In addition, approximately 32.54% of the weight loss of the porous calcium carbonate at 590–742 °C was attributed to the thermal decomposition of the calcium carbonate. After removing the weight loss of organic matter, the weight loss of the porous calcium carbonate due to calcium carbonate decomposition accounted for approximately 38.26% of the whole, which was 2.95% different from the theoretical weight loss of 44.00% of calcium carbonate. This was due to polyacrylic acid and sodium dodecyl sulfate decomposition residues in the porous calcium carbonate.

For the PAA and PCC (2 wt%)/PAA, the weight loss at 30–313 °C and 30–323 °C was mainly due to the removal of water and the –COOH groups [52]. For the PAA, an approximately 16.42% weight loss at 313–407 °C was attributed to the decomposition of the short or straight chains of polyacrylic acid [53]. As shown in Figure 4B, in the PCC (2 wt%)/PAA, the decomposition temperature of the polyacrylic acid chain was increased to 323 °C because the porous calcium carbonate provided additional crosslinking to the PCC (2 wt%)/PAA, enhancing the thermal stability. In addition, Figure 4A shows that, as the content of porous calcium carbonate in the PCC/PAA increased from 2 wt% to 8 wt%, the decomposition temperature range of short chains in the polyacrylic acid shrunk from 323–410 °C to 354–384 °C, and the weight loss also increased from 18.40% to 75.43%. The results showed that the porous calcium carbonate was a crosslinking site of the polyacrylic acid chain. With the increase in porous calcium carbonate content, the increase in crosslinking sites led to a decrease in the length of the polyacrylic acid chain, which led to an increase in the decomposition rate of the polyacrylic acid chain and an increase in weight loss.

As shown in Figure 4A, the weight loss of the PAA and PCC (2 wt%)/PAA at 407–553 °C and 410–530 °C was attributed to the decomposition of the crosslinked network [54]. For PCC/PAA, the decomposition of the crosslinked network ended at 530 °C, which was lower than that of PAA at 23 °C. The explanation was that the porous calcium carbonate weakened the hydrogen bond crosslinking of the network, leading to the premature completion of the polymer network decomposition. However, the weight loss attributed to the crosslinked network continuously decreased with increasing porous calcium carbonate content, which was attributed to the shortening of the length of the polyacrylic chain in the PCC/PAA due to the increase in the porous calcium carbonate content, and the stability of the crosslinked network structure reduced. The above results showed that the crosslinking between the porous calcium carbonate and polyacrylic acid chains enhanced the thermal stability of the PCC/PAA. However, as the content of porous calcium carbonate increased from 2 wt% to 8 wt%, the thermal stability of the PCC/PAA crosslinked network gradually decreased.

### 3.2. Crosslinking Mechanism between Porous Calcium Carbonate and Polymeric Chains

**Figure 5:**
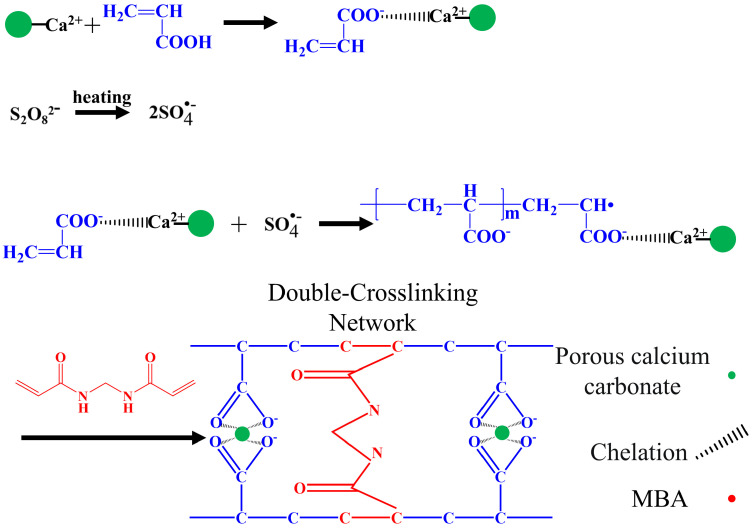
Crosslinking mechanism between porous calcium carbonate and polymeric chains.

Figure 5 shows the crosslinking mechanism of the porous calcium carbonate with the polymeric chain. First, the –COO^−^ in the acrylic acid chelated with Ca^2+^ on the surface of the porous calcium carbonate. Second, the persulfate produced sulfate anion radicals under heating, which were assigned to the acrylic acid monomer and formed polyacrylic acid by multidirectional grafting polymerization on the porous calcium carbonate surface [55]. Finally, during the polymerization of the linear polyacrylic acid molecules, the chemical crosslinker MBA formed a crosslinking network between the growing polyacrylic acid chains through the terminal vinyl group, and the porous calcium carbonate chelated with the –COO^−^ group in the polyacrylic acid chain through Ca^2+^, which also participated in the crosslinking of polyacrylic acid chains. The PCC/PAA had a double-crosslinked network structure constructed by the porous calcium carbonate and MBA.

### 3.3. Property Testing

#### 3.3.1. Water Absorption Test

The study in Section 3.1 found that the porous calcium carbonate affected the crosslinked network structure of the PCC/PAA. The water absorbency, water-retention rate, and reswelling water absorbency of the PAA and PCC/PAA were studied to understand the effect of the porous calcium carbonate on the performance of the PCC/PAA. A comparison of the maximum water absorbency of the superabsorbent with the addition of different inorganic fillers is summarized in Table 1. A maximum water absorbency in deionized water of 935 g/g and 0.9 wt% NaCl water solution of 80 g/g for the PCC/PAA were obtained in this work, which was higher than those in previous works, suggesting that the involvement of the porous calcium carbonate in the crosslinking process of the polymer through the Ca^2+^ site on the surface can give greater flexibility to the crosslinked network to enhance the water absorbency of the PCC/PAA.

**Table 1:** Comparison of water absorbency of PCC/PAA with other superabsorbents containing inorganic fillers.

Raw Materials	The Ratio of Monomers to the Inorganic Filler	Water Absorbency in Deionized Water (g/g)	Water Absorbency in 0.9 wt% NaCl (g/g)	Ref.
Acrylic acid, kaolin	Approximately 13:1	670	N/A	[56]
Acrylic acid, oil shale semicoke	Approximately 10: 1	420	65	[57]
Acrylic acid, bentonite clay	10:1	130	Approximately 20	[58]
Acrylic acid, montmorillonite	Approximately 7:1	400	N/A	[59]
Acrylic acid, acrylamide, montmorillonite,	Approximately 533:1	714	62	[60]
Acrylamide, montmorillonite	Approximately 15:1	721	Approximately 50	[61]
Acrylic acid, acrylamide, halloysite nanotube	20:1	537	N/A	[62]
PCC (2 wt%)/PAA	50:1	935	80	This work

**Figure 6:**
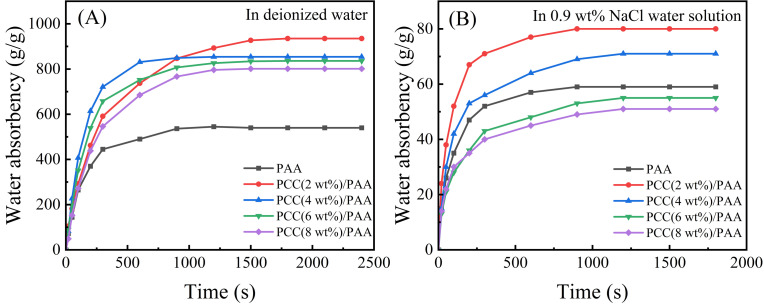
Water absorbency of PAA and PCC/PAA with 2–8 wt% porous calcium carbonate in deionized water (**A**) and 0.9 wt% NaCl water solution (**B**).

Figure 6A shows the water absorbency of the PAA and PCC/PAA with 2–8 wt% porous calcium carbonate in deionized water. Adding porous calcium carbonate can improve the water absorbency of the PCC/PAA, reaching 935 g/g for PCC (2 wt%)/PAA, which is higher than the 395 g/g for PAA. According to the FT-IR and XPS analysis, the reason was the crosslinking of the porous calcium carbonate with the polyacrylic chains. The entanglement between the polymer chains within the PCC/PAA weakened due to the crosslinking of the porous calcium carbonate, which resulted in more water storage space within the PCC/PAA and increased water absorbency [63]. In addition, acrylic acid can be grafted and copolymerized in multiple directions on the porous calcium carbonate surface due to the Ca^2+^ crosslink point, which makes the porous calcium carbonate act as a multidirectional crosslinking point inside the PCC/PAA. Polymer chains can carry tensile forces in multiple directions, so the crosslinked network of PCC/PAA is more flexible and can freely expand to improve the water absorbency. However, when the content of porous calcium carbonate increased from 2 wt% to 8 wt%, the water absorbency of the PCC/PAA decreased from 935 g/g to 801 g/g. With the increase in the content of porous calcium carbonate, on the one hand, the crosslinking points increased during the polymerization of the PCC/PAA, and the polyacrylic acid chain length that constituted the crosslinking network became shorter, which limited the free expansion of the crosslinking network. On the other hand, the relative content of the hydrophilic –COOH group in the crosslinked network decreased, and the water absorbency decreased. In addition, the absorption rate of the PCC/PAA increased with increasing porous calcium carbonate content in the first 900 s. The absorption rate was the fastest when the content of porous calcium carbonate reached 4 wt%, reaching saturation after 600 s in deionized water. According to the SEM images of the PCC/PAA, the PCC (4 wt%)PAA is rich in pores, which increases the contact area with water and, therefore, has the fastest absorption rate [64]. The above results indicated that the water absorbency of the PCC/PAA improved by the involvement of the porous calcium carbonate in the crosslinking of polyacrylic chains in the PCC/PAA.

Figure 6B shows the water absorbency of the PAA and PCC/PAA with 2–8 wt% porous calcium carbonate in the 0.9 wt% NaCl water solution. The water absorbency of the PAA in the 0.9 wt% NaCl water solution was much lower than that of deionized water because the high concentration of the salt solution would reduce the osmotic pressure difference between the polymer network and the external solution and the charge shielding effect of Na^+^ ions. As a result, the electrostatic repulsion between –COO^−^ in PAA is weakened [65], the network structure of PAA cannot be stretched freely, and water absorbency decreases significantly. However, in the 0.9 wt% NaCl water solution, the water absorbency of the PCC (2 wt%)/PAA increased from 60 g/g to 80 g/g. The SEM analysis results showed that, after the polyacrylic chain was crosslinked by the porous calcium carbonate, pores appeared in the PCC/PAA. When the network expansion was limited, these pores could still absorb water to improve salt tolerance. The salt tolerance of the PCC/PAA continued to decrease as the porous calcium carbonate content increased from 2 wt% to 8 wt%, attributed to the continuously reducing relative content of the –COOH groups of PCC/PAA. In conclusion, PCC/PAA has increased salt tolerance and will have greater application potential in medical and health products.

#### 3.3.2. Water Absorbency Test in Different pH Solutions and Different Salt Solutions

**Figure 7:**
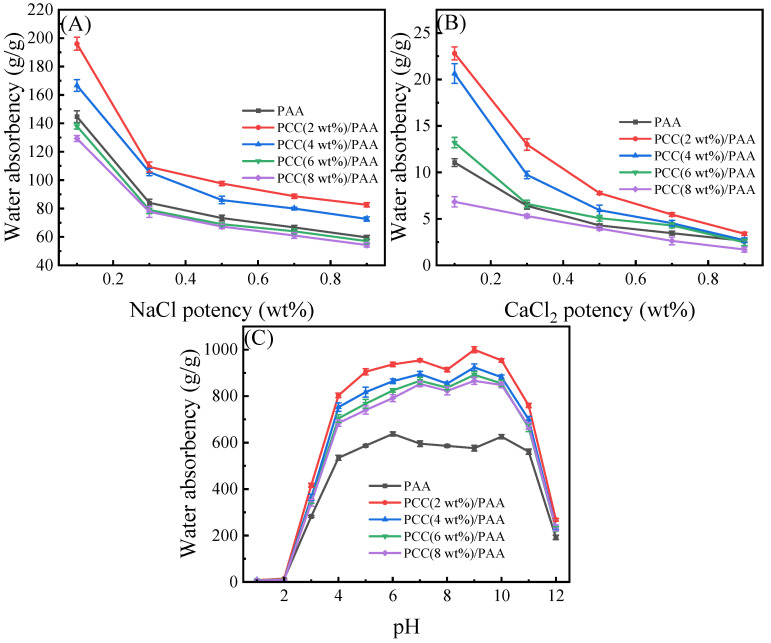
Water absorbency of PAA and PCC/PAA with 2–8 wt% porous calcium carbonate in NaCl (**A**), CaCl_2_ (**B**), and pH = 1–12 solutions (**C**).

Figure 7 shows the influence of the NaCl and CaCl_2_ solutions on the water absorbency of the PAA and PCC/PAA with 2–8 wt% porous calcium carbonate. As shown in Figure 7A, the water absorbency of the PAA decreased with increasing NaCl water solution concentration, which was due to the high concentration of salt solution reducing the osmotic pressure difference between the polymer network and the external solution, weakening the electrostatic repulsion between –COO^−^ and limiting the expansion of the PAA network. However, the crosslinked network structure of the PCC (2 wt%)/PAA and PCC (4 wt%)/PAA was improved by the porous calcium carbonate, and the water absorbency was higher than that of the PAA in the NaCl water solution. However, when the content of porous calcium carbonate was higher than 4 wt%, the excessive crosslinking points reduced the flexibility of the PCC/PAA crosslinking network and decreased the water absorbency of the PCC/PAA. As shown in Figure 7B, the water absorbency of the PAA further reduced as the ionic strength of the CaCl_2_ solution increased. The –COOH group in the PAA structure will interact with Ca^2+^ ions, leading to crosslinking of the polymer network and difficulty for water to enter the PAA interior. At this time, increasing the internal pores of PCC/PAA can effectively improve the water absorbency, so the water absorbency of the PCC(2 wt%)/PAA, PCC (4 wt%)/PAA, and PCC (6 wt%)/PAA was higher than that of the PAA. Especially in the 0.1 wt% CaCl_2_ solution, the water absorbency of the PCC (2 wt%)/PAA was two-times that of the PAA. This was because the pores in the PCC/PAA can increase the storage space so that water can still enter the interior of the PCC/PAA when the crosslinked network of the PCC/PAA has difficulty expanding in the salt solution, and the water absorbency of the PCC/PAA in the CaCl_2_ solutions increased. However, when the content of porous calcium carbonate was higher than 6 wt%, the crosslinked network structure with low flexibility reduced the water absorbency of the PCC/PAA.

An important characteristic of polyacrylic acid polymers is their ionizable –COOH groups, which leads to their pH-sensitive properties. The water absorbency of the PAA and PCC/PAA with 2–8 wt% porous calcium carbonate at pH = 1–12 solutions was evaluated. As shown in Figure 7C, the PAA and PCC/PAA showed pH sensitivity. Four stages can be observed for the PCC/PAA: (1) in the first stage, water absorbency increased as the pH increased from 1 to 7; (2) in the second stage, water absorbency declined with the further increase of the pH values up to 8; (3) in the third stage, water absorbency increased with the pH values increasing to 9; (4) in the fourth stage, water absorbency declined with the further increase of the pH values up to 12. The water absorbency of the PCC/PAA with 2–8 wt% porous calcium carbonate showed a similar trend as a function of the solution pH. However, the PAA showed a different water absorbency trend and obtained the maximum water absorbency at pH = 6.

The special water absorbency behavior of the PCC/PAA in different pH solutions was attributed to the protonation and deprotonation of –COOH and –COONa [66]. In acidic solutions, the formation of –COOH through the protonation of –COO^−^ will generate more hydrogen bonds between –COOH, which increases the physical crosslinking in the network and weakens the electrostatic repulsion in the –COOH. With increasing solution pH, –COOH groups deprotonate to –COO^−^ groups, which causes an increase in electrostatic repulsion, reduces the physical crosslinking of the polymer network, and increases water absorbency. The optimal water absorbency can be obtained when the solution is pH = 7. When pH = 8, the neutral solution is not conducive to –COOH group ionization, which is not conducive to expanding the network [67]. With the pH increasing from 8 to 9, the –COOH group ionization is enhanced, and the electrostatic repulsion between the –COO^−^ groups leads to high water absorbency. When the pH is higher than 9, electrostatic bonding between –COO^−^ groups and Na^+^ ions in alkaline solutions will confine the anion–anion repulsion among –COO^−^ groups and Na^+^ ions in the external solution, leading to a decrease in the osmotic pressure difference between the inner and outer sides of the crosslinked network and a decline in water absorbency. The difference in the trend of water absorbency between the PAA and PCC/PAA as a function of pH is attributed to the fact that, when porous calcium carbonate is used as the crosslinking site between polyacrylic chains, the relative length of the polyacrylic chain decreases and the ionization degree increases [68], so the ionization trend of the carboxylic acid group in PCC/PAA changes with the change in pH, resulting in the different pH sensitivities of PAA and PCC/PAA.

#### 3.3.3. Water-Retention-Rate Test

**Figure 8:**
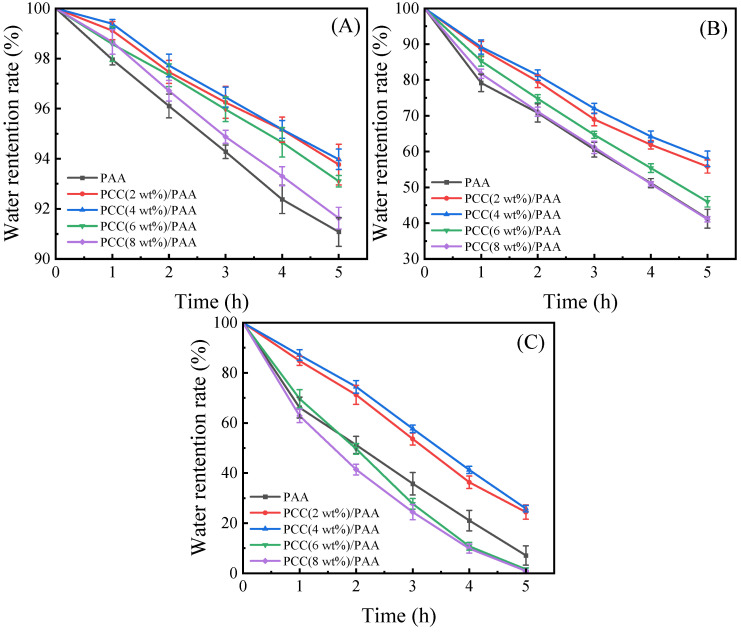
The water-retention rate at 20 °C (**A**), 40 °C (**B**), and 60 °C (**C**) of PAA and PCC/PAA with 2–8 wt% porous calcium carbonate.

Because of practical applications, water-retention capability is very important for superabsorbent polymers. Figure 8A–C show the water-retention ratio of the PAA and PCC/PAA with 2–8 wt% porous calcium carbonate. At 20 °C, the PCC/PAA with 2–6 wt% porous calcium carbonate retained approximately 93% of the water after 5 h of continuous observation, and the water-retention rate of the PAA was only approximately 91%. At 40 °C, the PCC/PAA with 2–4 wt% porous calcium carbonate could retain approximately 56–58% of the water after 5 h; when the temperature increased to 60 °C, there was roughly a 24–26% water-retention rate. Meanwhile, the water-retention rate of the PAA was only approximately 7%. From the FT-IR, XRD, and XPS analyses, it is clear that the porous calcium carbonate is involved in the crosslinking of the polymer network, forming a double-crosslinked network of PCC/PAA together with the chemical crosslinker MBA conformation. The TG analysis showed that the PCC/PAA had a stable crosslinking network structure so that water could be retained during the holding process within the PCC/PAA [69]. The rate of water loss is slowed by the hydrogen bonds formed between the –COOH groups in the crosslinked network and the water molecules [70]. However, when the content of porous calcium carbonate exceeded 4 wt%, the water-retention rate of the PCC/PAA reduced at 60 °C, which was attributed to the low-stability crosslinked network of the PCC/PAA with 4–8 wt% porous calcium carbonate being disrupted due to the aggravation of the movement of the polymer chain. Therefore, the water-retention rates of the PCC (6 wt%)/PAA and PCC (8 wt%)/PAA at 60 °C were lower than that of the PAA. In addition, the crosslinked network of the PAA and PCC (8 wt%)/PAA with low stability resulted in rapid water loss of the PAA and PCC (8 wt%)/PAA, which led to the tendency of the water-retention rate of the PAA to be the same as that of the PCC (8 wt%)/PAA.

#### 3.3.4. Reswelling Water Absorbency Test

**Figure 9:**
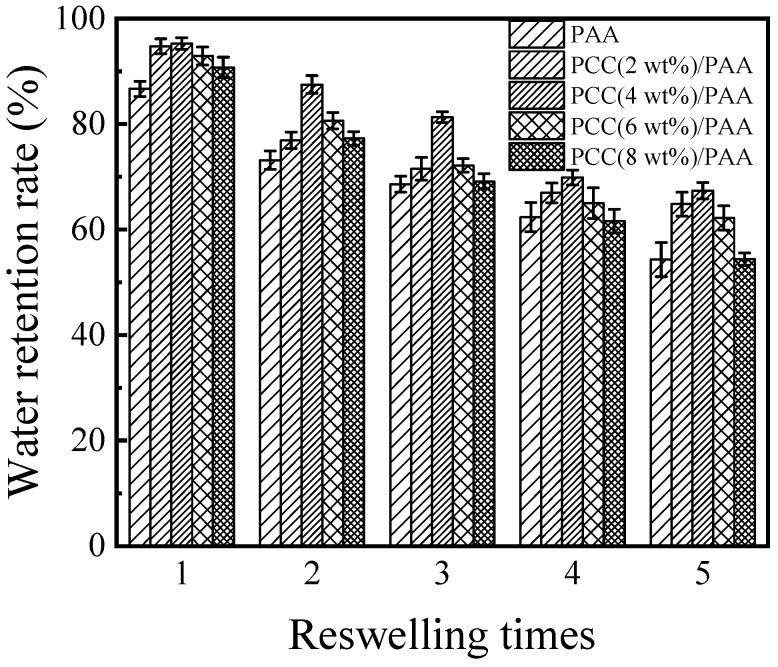
The reswelling water absorbency of PAA and PCC/PAA with 2–8 wt% porous calcium carbonate.

To evaluate the reswelling water absorbency of the PAA and PCC/PAA, consecutive swelling–deswelling cycles were carried out in deionized water at pH = 7 and 25 °C. The dry superabsorbent sample was immersed in excess deionized water to achieve swelling equilibrium. The swollen sample was dried in an oven at 60 °C and immersed again in excess deionized water to achieve swelling equilibrium. The loss of reswelling water absorbency of the superabsorbent is recorded in Figure 9. The results showed that the reswelling water absorbency of the PAA and PCC/PAA decreased with increasing reswelling time, which was due to the decomposition of weak crosslinking points in the crosslink network [71]. After five reswellings, the water absorbency of the PAA was only 54% of the first water absorbency. The reswelling water absorbency of the PCC/PAA significantly improved by adding porous calcium carbonate. The water absorbency of the PCC (4 wt%)/PAA achieved 67% of the initial water absorbency when it underwent five reswellings. Combining the results of FT-IR, XRD, and XPS, it is clear that the improved reswelling water absorbency of the PCC/PAA was due to the double-crosslinked network structure of the PCC/PAA constructed by porous calcium carbonate with MBA, which enabled the crosslinked network of PCC/PAA to undergo multiple reswelling water absorbencies without breaking [72]. However, as the content of the porous calcium carbonate increased from 4 wt% to 8 wt%, the reswelling water absorbency of the PCC/PAA decreased continuously. After five reswellings, the water absorbency of the PCC (8 wt%)/PAA was only 54% of the initial water absorbency. The decrease in reswelling water absorbency was attributed to the porous calcium carbonate as the crosslinking site of the polyacrylic acid chain. With increasing content, the crosslinking sites increased, which reduced the length of the polyacrylic acid chain and the flexibility of the crosslinking network [73], making the crosslinking network of the PCC/PAA easily destroyed in the process of reswelling water absorbency.

#### 3.3.5. Compressive Test

**Figure 10:**
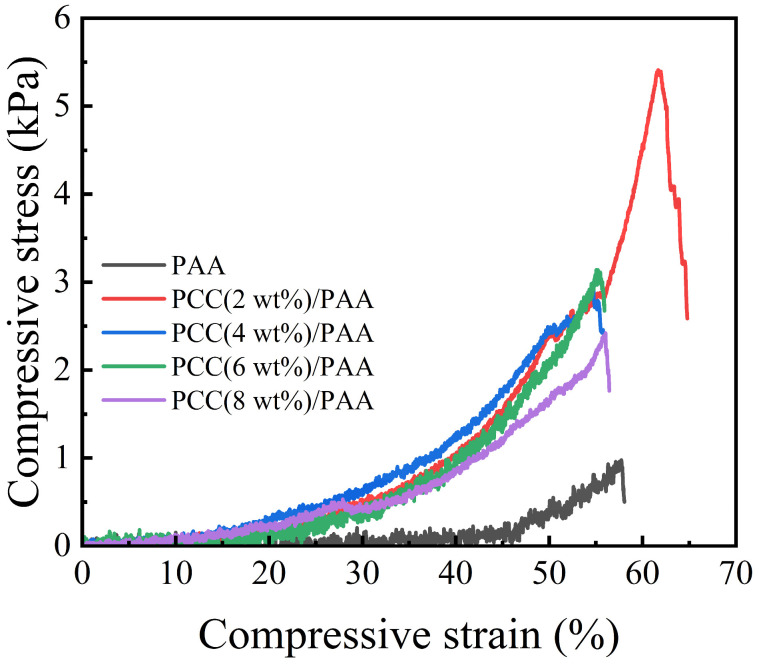
Compressive stress of PAA and PCC/PAA with 2–8 wt% porous calcium carbonate.

Figure 10 shows the compressive test of the PAA and PCC/PAA after water absorption. The compressive strength of the PAA was 1 kPa, which was the lowest among the samples. With the addition of the porous calcium carbonate, the compressive strength increased, and the compressive strength of the PCC (2 wt%)/PAA can reach approximately 5 kPa. This is because the porous calcium carbonate acts as a multidirectional crosslinking point inside the PCC/PAA, and each polymer chain can carry compressive stress in multiple directions and maintain the integrity of the crosslinked structure. As the content of porous calcium carbonate exceeded 2 wt%, the decreased mechanical properties of the PCC/PAA were attributed to the increase in the crosslinking sites, reducing the length of the polymer chain and decreasing the flexibility of the crosslinking network. The above results indicated that porous calcium carbonate can effectively improve the compressive strength of polyacrylic acid-based superabsorbents.

### 3.4. Mechanism of PCC/PAA Enhanced by Porous Calcium Carbonate

**Figure 11:**
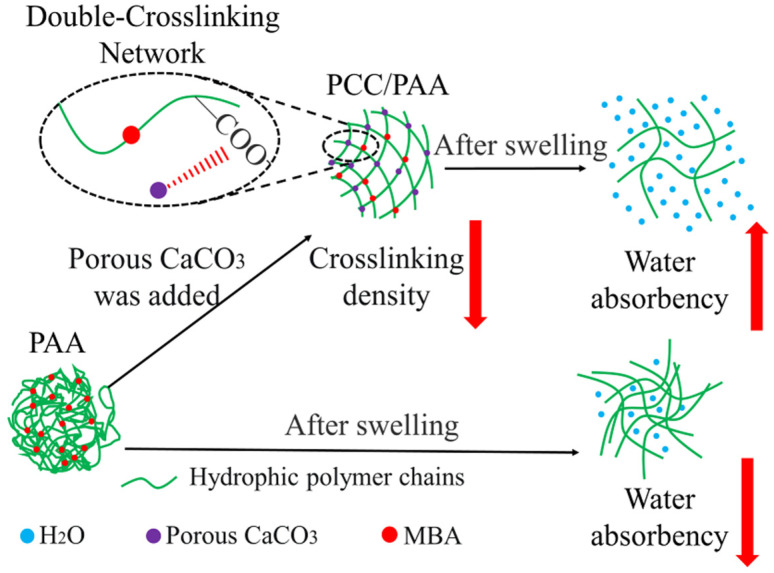
Mechanism of enhanced water absorbency and water-retention rate of PCC/PAA by porous calcium carbonate.

The mechanism by which porous calcium carbonate enhances the water absorbency and water-retention rate of PCC/PAA is shown in Figure 11. The Ca^2+^ site on the surface of porous calcium carbonate interacts with the –COOH group of acrylic acid through chelation so that acrylic acid copolymerizes on the surface of the porous calcium carbonate and acts as a crosslinking site between the polyacrylic acid chains. The crosslinking sites provided by the porous calcium carbonate reduce the chain entanglement caused by hydrogen bond interactions between the polyacrylic acid chains in PCC/PAA, resulting in the generation of pores in the crosslinking network to increase water storage space and improve water absorbency. Moreover, since porous calcium carbonate has a large specific surface area of 48.1 m^2^/g, which increases the starting point for the growth of the surface acrylic monomer chain, porous calcium carbonate can be used as a multidirectional crosslinking point in PCC/PAA to graft polyacrylic chains so that the polyacrylic chain can absorb tensile stress uniformly through a large number of molecular chains grafted on the surface of the porous calcium carbonate when it is fully stretched and make the crosslinking network more flexible. In addition, the combination of porous calcium carbonate and MBA forms a double-crosslinking network structure, which increases the stability of the crosslinking network so that the PCC/PAA cannot dissolve or break the crosslinking network after water absorption saturation, and the water-retention rate and reswelling water absorbency are improved.

## 4. Conclusions

A new composite superabsorbent PCC/PAA was obtained by copolymerizing porous calcium carbonate with an acrylic monomer. The porous calcium carbonate was prepared using natural minerals and had a specific surface area of 48.1 m^2^/g, which facilitated increased contact with the polymer. FT-IR characterization analysis showed that the C=O in the carboxylic acid group was redshifted by 10 cm^−1^ after the introduction of the porous calcium carbonate into the crosslinking network of the PCC/PAA, which confirmed that the porous calcium carbonate, as the crosslinking site of polyacrylic acid chains, was involved in the construction of the PCC/PAA crosslinking network by crosslinking polyacrylic acid chains. XPS analysis showed that the binding energy of C–O and C=O in the O 1*s* profile shifted to a higher energy level by 0.2 eV and 0.1–0.7 eV, respectively, after the involvement of the porous calcium carbonate in the crosslinking of the polyacrylic acid chains, confirming that porous-calcium-carbonate-crosslinked polyacrylic acid is based on chelation between the surface Ca^2+^ site and the –COOH group on the polyacrylic acid chain. The chelate crosslinking mode makes the porous calcium carbonate the multidirectional crosslinking point of the polyacrylic chain to achieve the simultaneous improvement of the water absorbency and water-retention rate of PCC/PAA, which was attributed to the rigid porous calcium carbonate reducing the entanglement between the polyacrylic acid chains, promoting the formation of pores inside the crosslinking network and increasing the water storage space. Compared with the rigid covalent crosslinking network introduced by the chemical crosslinker, the multidirectional crosslinking based on chelation allows the polyacrylic acid chain to absorb stress uniformly through a large number of molecular chains grafted on the porous calcium carbonate surface during the tensile process, making the crosslinking network more flexible to accommodate more water. The water absorbency of PCC/PAA with 2 wt% porous calcium carbonate in deionized water and 0.9 wt% NaCl water solution increased from 540 g/g and 60 g/g to 935 g/g and 80 g/g, respectively. The double-crosslinking network of porous calcium carbonate and the chemical crosslinker *N*,*N*’-methylene diacrylamide improved the stability of the PCC/PAA crosslinking network structure to ensure that the crosslinking network of the PCC/PAA will not dissolve or break after saturated water absorption, and the reswelling water absorbency and water-retention rate of the PCC/PAA improved. Compared with the water-retention rate of the superabsorbent without porous calcium carbonate, the water-retention rate of the PCC/PAA with 2 wt% porous calcium carbonate was increased by 244% after being kept at 60 °C for 5 h. The water absorbency increased by 19% after repeated use five times. In addition, the compressive strength of the PCC/PAA with 2 wt% porous calcium carbonate was approximately five-times higher than that of the superabsorbent without the porous calcium carbonate.

## Data Availability

The data presented in this study are available upon request from the corresponding author.
